# Utilization of Flavonoid Compounds from Bark and Wood. III. Application in Health Foods

**DOI:** 10.3390/molecules23081860

**Published:** 2018-07-26

**Authors:** Sosuke Ogawa, Yosuke Matsuo, Takashi Tanaka, Yoshikazu Yazaki

**Affiliations:** 1 Mimozax Co., Ltd., 4291-1, Miyauchi, Hatsukaichi-shi, Hiroshima 738-0034, Japan; 2 Department of Natural Product Chemistry, Graduate School of Biomedical Sciences, Nagasaki University, 1-14 Bunkyo-Machi, Nagasaki 852-8521, Japan; y-matsuo@nagasaki-u.ac.jp (Y.M.); t-tanaka@nagasaki-u.ac.jp (T.T.); 3 Department of Chemical Engineering, Monash University, Clayton, VIC 3800, Australia; yoshi.yazaki@monash.edu

**Keywords:** *Acacia mearnsii* bark, wattle tannin, proanthocyanidins, functional substance, health foods, foods with function claims

## Abstract

Dietary supplements ACAPOLIA^®^ and ACAPOLIA PLUS have been sold in Japan under the classification “Foods in General” for a number of years. In April 2015, the classification of “Foods with Function Claims” was introduced in Japan to make more products available to the public that were clearly labeled with functional claims based on scientific evidence. In order to obtain recognition of ACAPOLIA PLUS under this new classification, the following information needed to be established. The safety of the bark extract of *Acacia mearnsii* was shown from the history of the long-term safe consumption of the extract as a health supplement, together with several additional clinical safety tests. Robinetinidol-(4α,8)-catechin was detected by high performance liquid chromatography (HPLC) in the supplement and was suitable for use as the basis of the quantitative analysis. In clinical tests, the amount of change in the plasma glucose concentration in the initial 60 min after rice consumption by a test group who had been given the Acadia extract was significantly lower than the glucose concentration in the group that was given a placebo. The blood glucose incremental areas under the curve (IAUC) in the first 60 min after rice consumption were also significantly lower in the Acacia group. The functional mechanisms were explained in terms of the inhibition of the absorption of glucose in the small intestine and the reduction in the activity of the digestive enzymes caused by proanthocyanidins derived from *A. mearnsii* bark. As a result, ACAPOLIA PLUS was accepted as a “Food with Function Claims” in August 2016. ACAPOLIA PLUS is now sold under this new classification. The growth of a typical intestinal bacterium is inhibited by an extract containing flavonoid compounds from *A. mearnsii* bark; thus, one of the future directions of study must be a comprehensive investigation of the effect that flavonoid compounds, proanthocyanidins, have on intestinal bacteria.

## 1. Introduction

**Figure 1:**
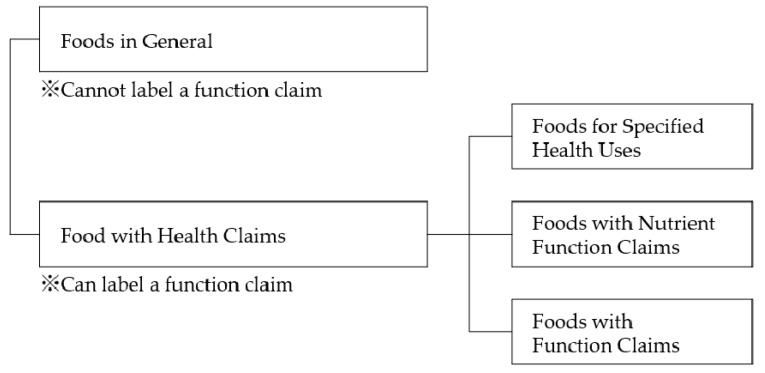
Categories of food products in Japan in April 2015.

Due to the strong antioxidant activity of flavonoid compounds, bark extracts from the French maritime pine (*Pinus pinaster*, synonym *P. maritima*) and radiata pine (*Pinus radiata*) have been commercialized as nutritional supplements. These commercial products are Pycnogenol and Enzogenol, respectively. The background and development of Pycnogenol and the basic difference in the preparation processes between Pycnogenol and Enzogenol have been described [1]. Due to the discovery that the superoxide scavenging activity (SOSA) value of wattle tannin is approximately ten times that of the extracts of the pine bark supplements, chemical, biochemical, and clinical studies on wattle tannin were conducted [1]. Wattle tannin has been developed as a nutritional supplement and marketed as ACAPOLIA^®^ in Japan since 2007. The details of these studies have been reported previously [1]. A further supplement, ACAPOLIA PLUS, which is ACAPOLIA^®^ with added vitamins has been sold since 2012 under the category of “Foods in General”, as shown in Figure 1. 

In Japan, the legal definition of “food” has been divided into “Foods in General” and “Foods with Health Claims”. A product classified as a “Foods in General” cannot be labeled with any health benefits. A product accepted under the category of “Foods with Health Claims” can be labeled with specified health claims or nutrient function claims. The advertisement of foods classed as “Foods in General” is strictly controlled. The classification “Foods in General” is the base level and cannot be described further. The next category, “Foods with Health Claims”, was divided, until recently, into “Foods for Specified Health Uses” and “Foods with Nutrient Function Claims”. In order to make more products that were clearly labeled with functional claims based on scientific evidence available to the general public, and to enable consumers to make more informed choices, an additional category “Foods with Function Claims” was introduced in April 2015, as shown in Figure 1 [2,3]. 

The acceptance of ACAPOLIA PLUS under the category “Foods with Function Claims” offers a commercial advantage. As a requirement, the following scientific tests were conducted and accepted by the Secretary-General of the Consumer Affairs Agency in Japan. 

1. The supplement safety has to be substantiated.

2. An analytical method for the qualitative and quantitative determination of the functional substance or substances must be established.

3. The effectiveness of the supplement must be substantiated.

4. The functional mechanisms must be fully explained.

This paper briefly describes the process whereby the health food supplement containing proanthocyanidins (flavonoids) derived from *Acacia mearnsii* bark came to be accepted in the “Foods with Function Claims” category for the control of blood glucose in humans.

## 2. Results

### 2.1. ACAPOLIA PLUS Safety

In order to substantiate the ACAPOLIA PLUS safety requirements, a history of consumption of *A. mearnsii* bark extract by humans and a safety test on functional substances (which in this case was proanthocyanidins-derived *A. mearnsii* bark) were assessed as described below.

As was previously indicated, ACAPOLIA^®^ has been on the market in Japan for more than ten years, and ACAPOLIA PLUS for a shorter period. Both supplements are manufactured by Mimozax Co., Ltd. (Hiroshima, Japan). The tablets of both ACAPOLIA^®^ and ACAPOLIA PLUS contain a daily dose of 250 mg of the extract. In the time that these flavonoid-containing supplements have been on the market, they have been consumed by more than 100,000 people. There have been no instances of serious adverse events reported by consumers to Mimozax Co., Ltd (Hiroshima, Japan). during this time. Therefore, it was concluded that a daily dose of 250 mg of the extract could be taken safely. However, additional specific safety tests were undertaken as described below.

Using healthy male adult subjects, four safety tests of tablets which contain proanthocyanidins derived from *A. mearnsii* bark as the functional substance were undertaken, see Table 1, and reported by Kataoka et al. [4]. Two of the tests were single-dose tests and the other two were 4-week repeated dose tests. The results of both the single-dose safety study and the 4-week repeated dose safety study showed no safety concerns for daily doses of up to 1000 mg of the extract. These results supported the safety of the product. 

These results were considered sufficient for the supplement safety requirement. However, if the Secretary-General of the Consumer Affairs Agency in Japan decided that the history of consumption by humans based on actual intake data was not satisfactory, further safety tests would be needed to show that the supplements were safe up to a level of five times the recommended daily intake. In order to satisfy the guideline and confirm the safety of the functional substance, additional overdose studies were undertaken in which a total of 29 healthy adult subjects were involved. The trials and results were reported by Ogawa et al. [5] in 2017. The subjects were randomized to an Acacia group (who each received 1875 mg of *A. mearnsii* bark extract per day) or a Placebo group (who received zero extract). The trial lasted for four weeks in a double-blind, parallel study. The results indicated that a daily intake of 1875 mg of an Acacia dietary supplement was safe for healthy adults. 

### 2.2. Qualitative and Quantitative Analyses of ACAPOLIA PLUS

In circumstances where the functional substance of a supplement is a natural product, it is necessary to identify the origin of the functional substance and the chemical equivalent of the functional substance. The *A. mearnsii* extract used as the raw material for ACAPOLIA PLUS has been produced as a food-grade substance in South Africa for a number of years; methods of qualitative and quantitative analyses of the functional substance of the supplement have been researched and confirmed as follows:

#### 2.2.1. Qualitative Analyses

**Figure 2:**
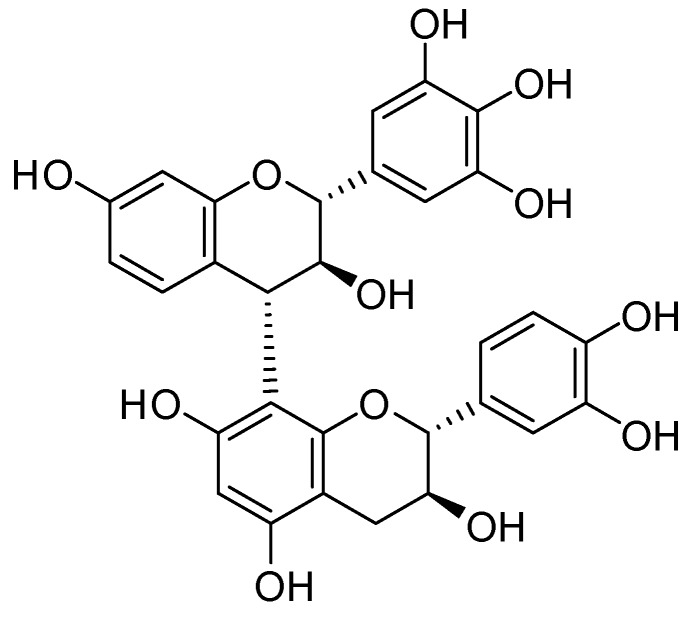
Structure of robinetinidol-(4α,8)-catechin.

**Figure 3:**
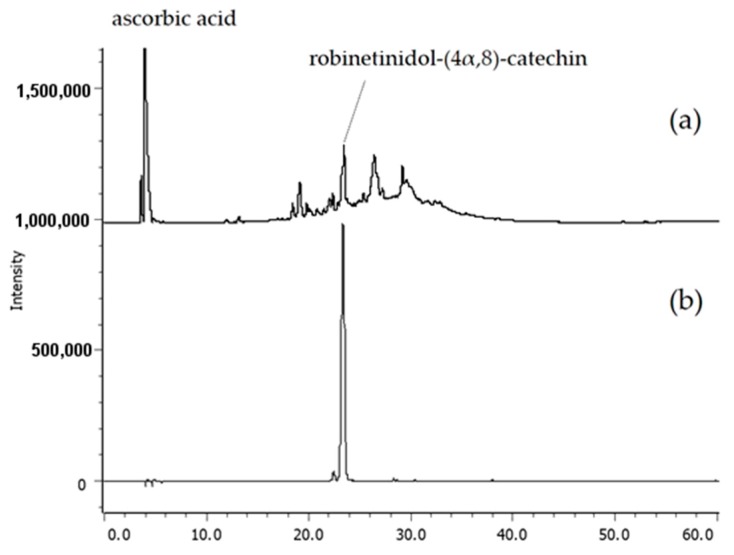
HPLC profiles of (**a**) ACAPOLIA PLUS (1 tablet/10 mL) and (**b**) robinetinidol-(4α,8)-catechin. Detection wavelength: 230 nm; injected volume: 10 μL for each sample.

Robinetinidol-(4α,8)-catechin, shown in Figure 2, was reported as being a constituent of *A. mearnsii* and, to date, has not been isolated from other plants [6,7,8,9,10]. Therefore, whether *A. mearnsii* extract is included in any preparation may be determined by establishing if robinetinidol-(4α,8)-catechin is present. The qualitative analysis for proanthocyanidins was performed by HPLC, as shown in Figure 3. The detailed method of qualitative analysis is described below.

One tablet containing *A. mearnsii* extract was powdered and extracted with 60% aqueous ethanol (2.0 mL). After filtration (membrane filter, 0.45 μL), 10 μL of filtrate was analyzed by HPLC. Robinetinidol-(4α,8)-catechin (prepared from *A. mearnsii* extract [10]) was also analyzed as a standard (*t*_R_ 23.1 min). Analytical HPLC was performed using a Cosmosil 5C_18_-ARII (Nacalai Tesque, Kyoto, Japan) column (250 × 4.6 mm, i.d.) with a gradient elution of 4–30% (39 min) and 30–75% (15 min) CH_3_CN in 50 mM H_3_PO_4_ at 35 °C (flow rate, 0.8 mL/min; detection, Jasco photodiode array detector MD-2010).

#### 2.2.2. Quantitative Analyses

The method for the quantitative analysis of proanthocyanidins was developed based on the Folin-Ciocalteu method. The Folin-Ciocalteu method is the major method used for the quantification of phenols and polyphenols [11]. However, other antioxidant compounds, such as ascorbic acid, also yield colored products when this method is used [12]. It is essential to remove them in order to accurately determine the amount of proanthocyanidins in the tablets. Therefore, after the powdered tablets were suspended in water and treated with ultrasound, the solution was filtered. The filtrate was applied to a C_18_ cartridge, and the non-adsorbed constituents (including ascorbic acid) were washed with water. No proanthocyanidins were detected in the water eluate. The adsorbed constituents including proanthocyanidins were eluted with methanol, and this solution was used for the quantitative estimation of the proanthocyanidins with the use of the Folin-Ciocalteu reagent. (+)-Catechin was used as a positive control, and the proanthocyanidins content was evaluated as a catechin equivalent. (+)-Catechin is commercially available and has a structure similar to those of the proanthocyanidins contained in *A. mearnsii* [10]. Therefore, accurate quantitative values reflecting the proanthocyanidins content can be obtained.

In order to increase the accuracy of the value of the proanthocyanidins content, the proanthocyanidins fraction prepared from *A. mearnsii* extract could be used as a positive control. However, the *A. mearnsii* extract contains phenolic compounds other than proanthocyanidins, such as monomer catechins [10]. Therefore, removing these from the quantitative values affords a more accurate estimate of the proanthocyanidins content. As a result of the quantitative analysis, six tablets of ACAPOLIA PLUS contain 120 mg (catechin-equivalent) or 163 mg (*A. mearnsii* proanthocyanidins-equivalent) of proanthocyanidins. A detailed method of the quantitative analysis is described below.

Tablets containing *A. mearnsii* extract were powdered using a mortar and pestle. Upon grinding, 100 mg of this powder was suspended in water using a 10 mL measuring flask, and treated with ultrasound for 3 min. The suspension was filtered through a membrane filter (0.45 μm), and the filtrate (1.0 mL) was applied to water-substituted Sep-Pak Plus C_18_ (Short, 360 mg/0.7 mL, Waters, Milford, MA, USA). The non-adsorbed constituents were eluted with water (2.0 mL), after which the adsorbed constituents containing the proanthocyanidins were eluted with 3.0 mL methanol. The methanol-eluted solution was diluted to 5 mL in a measuring flask, and water (1400 μL) was added to the diluted solution (600 μL). The resulting solution was diluted to concentrations of 50% and 25% with 30% aqueous methanol, and these solutions were subjected to quantitative analysis. The prepared sample solution or a standard solution (30% aqueous solution of (+)-catechin (Tokyo Chemical Industry Co., Tokyo, Japan) (50.0, 25.0, 12.5, 6.25 μg/1.0 mL)) (100 μL) was applied to a 96 well microplate. After which, 25 μL of Folin-Ciocalteu’s phenol reagent (Kanto Chemical Co., Tokyo, Japan) was added to each well and left to stand for 3 min. After standing, 25 μL of 10% aqueous sodium carbonate solution was added to each well and allowed to stand for 1 h with light shielding at room temperature to allow the color to develop. After one hour, the UV/vis absorbance was measured at 690 nm using an Emax precision microplate reader (Molecular Devices, San Jose, CA, USA).

### 2.3. Effectiveness Clinical Trial with ACAPOLIA PLUS on Suppressing Postprandial Blood Glucose Elevation

**Figure 4:**
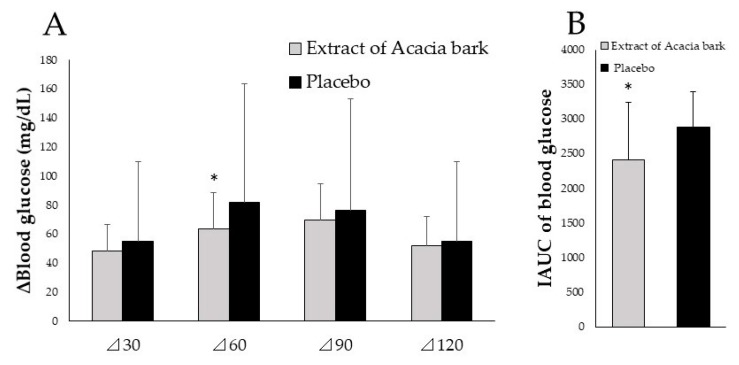
The amount of change in the plasma glucose concentration (**A**) and incremental area under the curve of blood glucose (0–60 min) (**B**) of non-diabetic individuals administered cooked rice. Each bar represents the mean for 13 non-diabetic individuals ± standard deviation. □ Extract of Acacia bark, ■ Placebo * Significantly different from placebo *p* < 0.05 (Students’ *t*-test)

The scientific evidence for the proposed functional claims must be explained by one of the following methods. The first is a clinical trial with a finished product, and the second is a systematic literature review on a finished product or functional substance. In this case, a clinical trial with the finished product, ACAPOLIA PLUS, was conducted and reported by Takeda et al. [13] in 2016. Subjects in this study were as follows; (1) non‒diabetic men and women aged between 40 and 60 years of age; and (2) possessed a fasting plasma glucose (FPG) level of between 110‒125 mg/dL or an impaired glucose tolerance (120 min glucose level in a 75 g oral glucose tolerance test (OGTT), between 140‒199 mg/dL). A total of 13 subjects were finally selected for this study. The changes in the blood glucose levels were monitored over a period of 120 min after taking ACAPOLIA PLUS or a placebo before consuming 200 g of cooked rice. The results showed that the amount of change in the plasma glucose concentration in the initial 60 min after rice consumption in the Acacia group (the group given ACAPOLIA PLUS) was statistically lower than the group that was given a placebo, see Figure 4A. The blood glucose incremental postprandial areas under the curve (IAUC), shown in Figure 4B, in the initial 60 min after the consumption of rice were also significantly lower in the Acacia group. This study was conducted with relatively few participants and therefore should be repeated with larger numbers of participants. However, from the limited study, it was concluded that the ACAPOLIA PLUS containing proanthocyanidins derived from *A. mearnsii* bark did reduce the absorption of carbohydrates and limited the rise of blood glucose in humans.

### 2.4. Functional Mechanisms

The functional mechanisms for the reduction in the carbohydrate absorption and the limit of the blood glucose rise in humans after taking the extract of *A. mearnsii* bark were explained in the following terms of two potential mechanisms. Namely, the inhibition of the absorption of glucose in the small intestine by the bark extract and the reduction in the digestive enzyme activity by the extract.

Ikarashi et al. [14] revealed that the extract significantly inhibited the increase in plasma glucose concentration after maltose, sucrose, or glucose loading in vivo. This inhibitory effect may be attributable to the inhibition of glucose uptake via a sodium-dependent glucose transporter (SGLT) and glucose transporter (GLUT) in the small intestine. It has been reported that there is inhibition of digestive enzymes such as lipase [10,14], α-amylase [10,15], and glucosidase [14] by the extract from *A. mearnsii* bark. Kusano et al. [10] revealed that proanthocyanidins from the bark of *A. mearnsii* exhibited strong α-amylase inhibition activity, comparable to the results seen with black tea and much stronger than those seen with green tea, oolong tea, or guava leaf extracts. The proanthocyanidins exhibited strong lipase inhibition activity. The most active proanthocyanidins fraction was characterized by spectroscopic and chemical methods and shown to contain tetrameric to octameric compounds mainly composed of robinetinidol units. Ikarashi et al. [14] revealed that the extract of *A. mearnsii* bark inhibited the activity of lipase, maltase, and sucrase. Matsuo et al. [15] reported that the hot-water bark extracts were carefully fractionated into six fractions using Diaion HP20 column chromatography with monitoring using thin-layer chromatography (TLC) employing two different solvent systems. The fractions (2–6) contained phenolic compounds and exhibited α-amylase inhibitory activity. Contrastingly, the fraction 1 which contained sugars and polyols did not exhibit any α-amylase inhibitory activity. Spectroscopic results clearly indicated that fractions with strong inhibitory activity contained proanthocyanidins oligomers with catechol-type B-rings rather than pyrogallol-type B-rings. HPLC analysis of the pyrolysis products showed peaks for pyrocatechol were only observed in the mixtures obtained from the fractions with high inhibitory activities.

In a clinical test, the ACAPOLIA PLUS reduced the absorption of carbohydrates and limited the rise of blood glucose in humans. Inhibition of the increase in the plasma glucose concentration by proanthocyanidins derived from *A. mearnsii* in vivo, and α-amylase and glucosidase inhibition activities by proanthocyanidins derived from *A. mearnsii* bark in vitro were considered as the functional mechanisms.

## 3. Discussion

### 3.1. ACAPOLIA PLUS Containing Flavonoid Compounds as a “Food with Function Claims”

The supplement safety has been confirmed, an accurate analytical method for the qualitative and quantitative determination of the functional substance has been developed, the supplement effectiveness has been confirmed, and the functional mechanisms explained all conditions required for the reclassification of ACAPOLIA PLUS. These data were submitted to the Secretary-General of the Consumer Affairs Agency for classification under “Foods with Function Claims” [2] and were accepted in August 2016. Sales of ACAPOLIA PLUS have commenced with a new label detailing the following functions. ACAPOLIA PLUS contains 163 mg of proanthocyanidins derived from Acacia bark as the functional substance in each daily dose, and the supplement has functions that reduce the absorption of carbohydrates and limit the postprandial rise of blood glucose in humans for a person who has a high blood glucose level or an easily increased blood glucose level.

### 3.2. Further Research of Biological Activities Based on a Microbiome Changed by Flavonoids

The effectiveness of clinical trials for the product containing proanthocyanidins of *A. mearnsii* bark in suppressing postprandial blood sugar elevation and the mechanism by which this occurs have been described. The bark extract inhibited the absorption of glucose in the small intestine and reduced the activity of the digestive enzymes in the digestive tract. It is believed that the bark extract functions in the digestive tract without being absorbed in the body. The question to be asked is, does the bark extract produce another effect in the small and large intestine or not? The answer to this question may lead to additional applications in health foods as “Food with Function Claims”. In addition, upon consideration of the other possibilities for the use of proanthocyanidins of *A. mearnsii* bark in dietary supplements, Ogawa et al. [16] reviewed the biological activities in a previous paper in 2018. According to that review, as the growth of a typical intestinal bacterium such as *Escherichia coli*, *Klebsiella pneumoniae*, *Proteus vulgaris*, and *Serratia marcescens* was inhibited by the extract from *A. mearnsii* bark, further study must investigate the effect that proanthocyanidins had on intestinal bacteria. Several papers reported the relationships between flavonoids and microbiome as follows. Goto et al. [17] reported that the effects of tea catechins on fecal contents and metabolites of elderly people. Tea catechins (300 mg), which were divided into three doses per day, were a meal supplement each day for six weeks. The tea catechins extract contain 62% catechins, including (−)-epicatechin (6.5%), (−)-epigallocatechin (18.5%), (−)-epicatechin gallate (7.0%), and (−)-epigallocatechin gallate (EGCG) (30.5%). In the same clinical test, Hara [18] reported that the amounts of *Lactobacillus* and *Bifidobacteria* referred to as “beneficial bacteria” increased, and the quantities of *Clostridium*, *Bacteroides*, and *E. coli* referred to as “bad bacteria” decreased while tea catechin was taken. In another cinical test, Okubo et al. [19], investigated the effects of tea polyphenol intake (0.4 g/volunteer, three times per day, for four weeks) on the fecal microflora, the bacterial metabolites, and pH using eight healthy human volunteers. Counts of *Clostridium* spp. significantly decreased during the tea polyphenol intake, which contains (+)-catechin, (−)-epicatechin, (+)-gallocatechin, (−)-epigallocatechin, (−)-epicatechin gallate, (−)-gallocatechin gallate, and (−)-epigallocatechin gallate. Unno et al. [20] also reported that EGCG inhibited the growth of *Clostridium* groups in vivo. 

Research has indicated that decreasing the level of the intestinal bacteria, *Clostridium* spp., by treatment with EGCG affects the biological activity of the host animals. Ikarashi et al. [21] revealed that the intake of a high-dose of green tea polyphenols results in a liver-specific decrease in the drug-metabolizing enzyme cytochrome P450 3A (CYP3A) expression level during an in vivo test. Ikarashi et al. [22] evaluated the liver-specific decrease in the CYP3A expression level observed after a high-dose intake of EGCG. Owing to the fact that EGCG, which is not absorbed in the intestine, causes a decrease in the level of lithocholic acid (LCA)-producing bacteria of *Clostridium* cluster XIVa in the colon, the level of LCA in the liver decreases, resulting in a decrease in the nuclear translocation of pregnane X receptor (PXR), which in turn leads to the observed decrease in the expression level of CYP3A. 

As not all the effects of proanthocyanidins derived from *A. mearnsii* bark on the intestinal bacteria of host animals have been studied, biological activities focused on the connection between the intestinal bacteria and the bark material must be determined. In the future, the relationships between proanthocyanidins derived from *A. mearnsii* bark and intestinal bacteria will likely be discovered, at which time the biological activities for host animals will be fully disclosed.

## 4. Conclusions

This paper briefly describes the process whereby the health food supplement ACAPOLIA PLUS, containing proanthocyanidins (flavonoids) derived from *A. mearnsii* as the functional substance, became approved under the “Foods with Function Claims” legislation for the control of blood glucose in humans. The following were revealed. The safety of the bark extract of *A. mearnsii* was established from the history of the safe consumption of the extract when taken as a health supplement and several additional clinical safety tests. Robinetinidol-(4α,8)-catechin in the supplement was detected by HPLC, and quantitative analyses detected 120 mg (catechin-equivalent) or 163 mg (*A. mearnsii* proanthocyanidins-equivalent) of proanthocyanidins in six tablets of the supplement. In the clinical test, the amount of change in the plasma glucose concentration in the initial 60 min after rice consumption in the Acacia group decreased significantly compared to the reduction found in the Placebo group. The blood glucose IAUC in the initial 60 min was significantly lower in the Acacia group. The functional mechanisms for the reduction in the absorption of carbohydrates and the limiting of the blood glucose rise in humans after taking the extract were explained in terms of the inhibition of the absorption of glucose in the small intestine and the reduction in the digestive enzymes activity by proanthocyanidins derived from *A. mearnsii* bark.

A potential further direction for the proanthocyanidins (flavonoids) in other supplements with new functional claims was discussed. As it is known that the growth of a typical intestinal bacterium is inhibited by the extract from *A. mearnsii* bark, and the possibilities for the effective use of the flavonoid compounds, proanthocyanidins, on the control of intestinal bacteria were briefly discussed.

## Figures and Tables

**Table 1:** Clinical test conditions of four different tests.

	Test No.	Clinical Test Conditions
1*	Study Ⅰ	Twenty subjects were divided to 4 groups of 5 subjects and took study diets of 2, 4, 6 and 8 tablets (each 250, 500, 750 and 1000 mg as the *A. mearnsii* bark extract) during a single trial.
	Study Ⅱ	Five subjects took study diets of 12 tablets (1500 mg as the *A. mearnsii* bark extract) during a single trial.
2*	Study Ⅲ	Twenty-two subjects were divided into 2 groups and each group took a study diet of either 2 tablets (a daily total of 250 mg of the *A. mearnsii* bark extract) or 4 tablets (a daily total of 500 mg of the *A. mearnsii* bark extract) over a 4-week trial period.
	Study Ⅳ	Twenty-five subjects were divided into 2 groups and each group took a study diet of either 6 tablets (a daily total of 750 mg of the *A. mearnsii* bark extract) or 8 tablets (a daily total of 1000 mg as the *A. mearnsii* bark extract) over a 4-week trial period.

1*: single-dose test, 2*: 4-week repeated dose safety test.
